# Spatially reconfigurable antiferromagnetic states in topologically rich free-standing nanomembranes

**DOI:** 10.1038/s41563-024-01806-2

**Published:** 2024-02-19

**Authors:** Hariom Jani, Jack Harrison, Sonu Hooda, Saurav Prakash, Proloy Nandi, Junxiong Hu, Zhiyang Zeng, Jheng-Cyuan Lin, Charles Godfrey, Ganesh ji Omar, Tim A. Butcher, Jörg Raabe, Simone Finizio, Aaron Voon-Yew Thean, A. Ariando, Paolo G. Radaelli

**Affiliations:** 1 Clarendon Laboratory, Department of Physics, University of Oxford, Oxford, UK; 2 Department of Physics, National University of Singapore, Singapore, Singapore; 3 Department of Electrical and Computer Engineering, National University of Singapore, Singapore, Singapore; 4 Swiss Light Source, Paul Scherrer Institut, Villigen, Switzerland; 5 Integrative Sciences and Engineering Programme, National University of Singapore, Singapore, Singapore

**Keywords:** Phase transitions and critical phenomena, Topological defects, Imaging techniques, Spintronics, Magnetic properties and materials

## Abstract

Antiferromagnets hosting real-space topological textures are promising platforms to model fundamental ultrafast phenomena and explore spintronics. However, they have only been epitaxially fabricated on specific symmetry-matched substrates, thereby preserving their intrinsic magneto-crystalline order. This curtails their integration with dissimilar supports, restricting the scope of fundamental and applied investigations. Here we circumvent this limitation by designing detachable crystalline antiferromagnetic nanomembranes of α-Fe_2_O_3_. First, we show—via transmission-based antiferromagnetic vector mapping—that flat nanomembranes host a spin-reorientation transition and rich topological phenomenology. Second, we exploit their extreme flexibility to demonstrate the reconfiguration of antiferromagnetic states across three-dimensional membrane folds resulting from flexure-induced strains. Finally, we combine these developments using a controlled manipulator to realize the strain-driven non-thermal generation of topological textures at room temperature. The integration of such free-standing antiferromagnetic layers with flat/curved nanostructures could enable spin texture designs via magnetoelastic/geometric effects in the quasi-static and dynamical regimes, opening new explorations into curvilinear antiferromagnetism and unconventional computing.

## Abstract (web summary)

Topological antiferromagnetic states are generated and spatially reconfigured in free-standing crystalline membranes of haematite through strain design.

## Main

Topological textures in antiferromagnetic (AFM) materials are whirling structures with spins oppositely aligned between two ferromagnetic (FM) sublattices. Beyond topological protection, this spin configuration affords unique benefits not enjoyed by their FM counterparts, including robustness against external perturbations, size scalability and ultrafast dynamics^1–5^. In fact, some topological AFM textures are predicted to exhibit spintronic analogues of relativistic physics, where their speed limit is set by the magnon group velocity^2,6^. This immense potential has resulted in a surge of interest in topological AFM states^1,3,7–9^.

Central to nucleating and controlling topological textures are various magneto-crystalline interactions, namely, anisotropy, exchange or spin–orbit torques. AFM systems, thus far, reported to host topological order^10–14^, for example, α-Fe_2_O_3_, CuMnAs and MnSc_2_S_4_, either were bulk crystals^14^ or were epitaxially grown on symmetry-matched crystalline substrates through advanced fabrication^10,12,13^. This markedly restricts their utility and flexibility compared with typical FM-based topological-texture-hosting metallic heterostructures, which are polycrystalline and can be simply grown by sputter deposition^1,8^. Therefore, further exploration and exploitation of topological AFM textures necessitate the development of crystalline AFM layers that can be integrated with dissimilar supports, for example, silicon or even non-crystalline flexible substrates.

We drew inspiration from recent developments in crystalline quantum material membranes, namely, free-standing crystals of macroscopic lateral dimensions with a thickness of ∼1–100 nm (refs. ^15–19^). These membranes are a relatively new form of crystalline matter occupying an intermediate position between bulk and two-dimensional materials, whilst having properties distinct from both. Generally, they have bulk-like magnetic/electronic properties but—akin to two-dimensional materials—are flexible and can withstand extreme deformations without undergoing fracture^16,17^. They can also be transferred post-growth to any desirable host, enhancing the ability to stack and twist complex heterostructures^18^.

Here we design and fabricate AFM nanomembranes that preserve the all-important magneto-crystalline interactions post-delamination. Having developed a scanning transmission X-ray microscopy (STXM)-based Néel vector reconstruction technique to image the local AFM order, we show that our detached membranes host a multichiral family of topological AFM textures, analogous to the Kibble–Zurek-like phenomenology previously observed in attached epitaxial films^10^. Moreover, we demonstrate a striking isothermal reconfiguration of local AFM background states, hosting these topological textures, across three-dimensional membrane ‘folds’. We present mechanical models confirming that our observations are consistent with the magneto-structural effects expected from flexure-based strains. Finally, we marry these advances by straining membranes in a gas-cell manipulator to realize an isothermal strain-driven topological transition. Our results pave the way for the development of AFM spintronics platforms exploiting membrane tunability via geometry and strain.

## Membrane design and fabrication

**Fig. 1:**
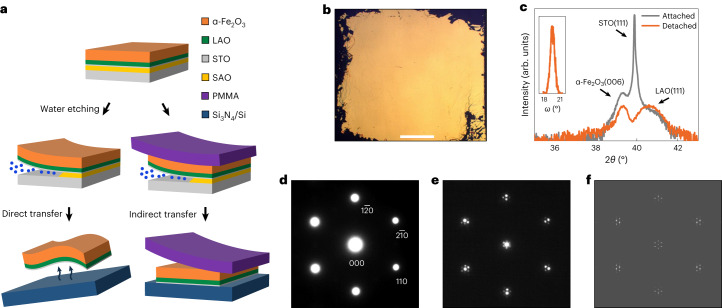
Membrane design and characterization. **a**, Free-standing membranes are prepared by the selective water etching of SAO (yellow), followed by the direct/indirect transfer of membranes (orange) onto desired silicon (Si) or silicon nitride (Si_3_N_4_) supports (blue). Indirect transfer requires an intermediate support such as PMMA (purple) to hold the membranes after water etching. For type-C membranes, a buffer layer made of LAO (green) and ultrathin STO (grey) was also grown (Methods). Layer thickness is not to scale. **b**, Large-area optical image of a buffered α-Fe_2_O_3_ membrane transferred onto Si. Scale bar, 1 mm. **c**, XRD (2*θ*–*ω* scans) of as-grown α-Fe_2_O_3_|LAO|STO|SAO film on an STO substrate (grey curve) and detached α-Fe_2_O_3_|LAO|STO membrane on a SiO_2_/Si substrate (orange curve). The out-of-plane (006) Bragg peak of α-Fe_2_O_3_ lies in the proximity of the (111) LAO buffer and (111) STO substrate peaks. The STO layer in the buffer is too thin to contribute a sizable signal in the detached sample. The inset displays the rocking curve (*ω* scan) of the detached membrane, exhibiting a full-width at half-maximum of ∼1.1°. **d**,**e**, SAED patterns of free-standing unbuffered (type-B) (**d**) and buffered (type-C) (**e**) α-Fe_2_O_3_ membranes obtained with an electron beam incident along the crystallographic *c* axis. **f**, Simulated SAED pattern of the type-C membrane (Methods) corresponding to the pattern in **e**. The simulation confirms that the satellite peaks in **e** emerge due to a lattice-mismatch moiré pattern^24,25^, resulting from the electron-beam interference across α-Fe_2_O_3_ and LAO lattices in the buffered membrane.

We fabricated free-standing membranes of (001)-oriented, Rh-doped α-Fe_2_O_3_ (referred to as α-Fe_2_O_3_ hereafter) using the selective water-etching technique (Methods) on epitaxial heterostructures grown by pulsed laser deposition^10,11,20^. The quality of α-Fe_2_O_3_ layers was found to critically depend on the choice of substrate and intermediate (buffer) layers to reduce interlayer lattice mismatch. Due to the trigonal symmetry of α-Fe_2_O_3_ (space group $R\bar{3}c$), we chose single-crystalline (001)-oriented α-Al_2_O_3_ and (111)-oriented SrTiO_3_ (STO) substrates as the growth templates, and (111)-oriented Sr_3_Al_2_O_6_ (SAO) as the water-soluble sacrificial layer^15,21,22^. The water etching of SAO resulted in free-standing oxide membranes, which were shifted to the desired support via either direct or indirect transfer (Fig. 1a). The former involves the direct scooping of the afloat membrane onto the support, whereas the latter requires the spin coating of a temporary organic support to hold the delaminated membrane before its final transfer. We have used both approaches for different experiments throughout this work.

We found that the direct growth of α-Fe_2_O_3_|SAO on (001)-oriented α-Al_2_O_3_ substrates (sample type A) results in oriented polycrystalline samples due to the large lattice mismatch between the various layers (Supplementary Section 1). The film quality particularly improves when α-Fe_2_O_3_|SAO is grown on (111)-oriented STO substrates (sample type B) due to the substantially lower mismatch between SAO and STO (in this orientation in bulk, *a*_SAO_/4 ≈ 5.60 Å and *a*_STO_ ≈ 5.51 Å), although the resulting α-Fe_2_O_3_ itself remains fairly defective. To improve the sample quality further, we added an intermediate buffer consisting of an ultrathin (111) STO layer followed by a thicker (111) LaAlO_3_ (LAO) layer between SAO and α-Fe_2_O_3_ (sample type C) (Methods and Supplementary Section 1). The thickness of the α-Fe_2_O_3_, LAO and STO layers were ∼30, 10 and 3 nm, respectively. Here LAO acts as a good buffer as it has a slightly smaller lattice constant (*a*_LAO_ ≈ 5.35 Å), reducing the mismatch with α-Fe_2_O_3_ (${a}_{{{\rm{Fe}}}_{2}{{\rm{O}}}_{3}}$ ≈ 5.03 Å), as well as being structurally close to both STO and SAO^21^. Moreover, the ultrathin STO increases the overall crystallinity^21^ and is found to be critical in aiding the delamination of overlayers in our buffered heterostructures. The addition of buffer layers results in free-standing AFM membranes with much larger crack-free areas compared with the unbuffered counterparts (Fig. 1b and Supplementary Section 1). Last, the control over sample quality and yield of these detachable membranes is superior to haematite layers prepared via the chemical exfoliation of natural iron-ore powder^23^.

The quality and orientation of our buffered α-Fe_2_O_3_ crystal membranes were ascertained by X-ray diffraction (XRD) and selected-area electron diffraction (SAED) (Fig. 1c–f and Supplementary Section 1). A unique feature of buffered membranes is the formation of a moiré pattern evident in the reciprocal space as satellite peaks in the SAED data. This is expected to be a ‘mismatch’ moiré pattern^24,25^, which results from electron-beam interference through the slightly mismatched lattices of α-Fe_2_O_3_ and buffer layers (Supplementary Section 10). This is validated by our diffraction simulation, which closely reproduces the experimental pattern (Fig. 1f). The resulting periodic perturbation at the α-Fe_2_O_3_–buffer interface does not appear to affect the magnetic properties of α-Fe_2_O_3_, as the length scales we study in magnetometry and X-ray microscopy, as well as the membrane thickness, are substantially larger than those of the mismatch pattern.

## Magnetic transition in membranes

The reliable generation of topological textures in α-Fe_2_O_3_ requires the presence of a spin-reorientation (Morin) phase transition, which mimics the Kibble–Zurek phenomenology^10^. At the Morin transition temperature *T*_M_, the anisotropy undergoes a sign reversal from easy axis (*K* > 0) to easy plane (*K* < 0) (refs. ^10,20^), causing spins to flip from out-of-plane (OOP) to in-plane (IP) configurations. The presence of a sharp Morin transition in the proximity of room temperature was confirmed by superconducting quantum interference device magnetometry^10,20^ as well as X-ray spectroscopy (Supplementary Section 1). This is in stark contrast to chemically exfoliated hematene membranes, where the Morin transition is completely suppressed due to the alteration of magneto-crystalline interactions^23^. Crucially, the transition in our detached membranes is qualitatively similar to those reported in the attached epitaxial films^10,11,20^, despite the former being more defective than the latter, with transitions in buffered α-Fe_2_O_3_ being particularly sharp (Supplementary Section 1). We conclude that our water-etched membranes are good free-standing platforms to seek out real-space topological AFM order.

## Nanoscale mapping of the AFM order

**Fig. 2:**
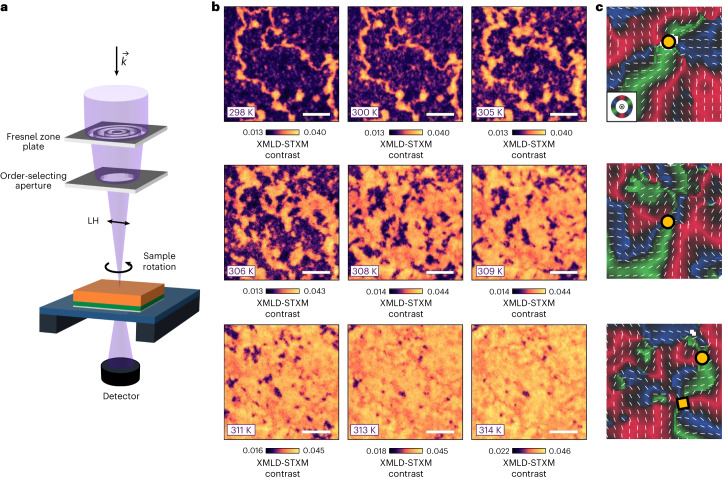
Morin transition and generation of topological AFM textures. **a**, Geometry of the STXM measurement, performed using linearly polarized X-rays ($\overrightarrow{k}$) that are normally incident onto the sample. **b**, Temperature evolution of the AFM STXM contrast obtained at the Fe L_3_-edge whilst warming the buffered membrane (type C) across *T*_M_, in the temperature range of 298–314 K. The OOP and IP contrasts are indicated in purple and yellow/orange, respectively. Scale bars, 2 µm. All the images were recorded at the same position. The energy-contrast scale was slightly varied across the transition to aid visualization^10^. **c**, Néel vector maps performed at 314 K (*T* > *T*_M_), produced by rotating the sample azimuth shown in **a**. The R–G–B colours (key inset in **c**, top) and thin white bars represent the IP AFM orientations. The white regions represent OOP orientations, whereas the black regions highlight the IP AFM directions, substantially deviating from the R–G–B directions. The yellow circles and squares indicate AFM merons and antimerons, respectively. The width of the images is ∼1.5 μm.

To image the local AFM textures, we performed STXM in the X-ray magnetic linear dichroism (XMLD) modality (Methods)—an element-specific spectro-microscopy technique, with a large depth of focus, which enables the unambiguous identification of AFM contrast. In XMLD-STXM, a beam of Fe L_3_-edge X-rays is focused onto the AFM membranes at normal incidence, whereas changes in absorption are monitored in transmission by a point detector (Fig. 2a). In this geometry, the X-ray polarization (linear horizontal (LH)) is in the basal plane of the α-Fe_2_O_3_ membranes, and IP and OOP AFM orientations are clearly distinguished as they contribute different XMLD signals^10^. Moreover, by varying the sample azimuth using an in situ rotation stage (PolLux beamline) or the X-ray polarization (SIM beamline), we systematically changed the relative orientation of the X-ray polarization and IP Néel order, enabling the nanoscale reconstruction of the AFM order^10,26,27^. Akin to our previous work with XMLD photoemission microscopy^10,26^, the XMLD-STXM contrast can resolve the IP AFM directions but cannot distinguish the absolute sign of the AFM order. Nevertheless, we can clearly identify topological textures in our membranes from these reconstructions.

## Evolution across the Morin transition

XMLD-STXM reveals that our α-Fe_2_O_3_ membranes host magnetic textures similar to those seen in attached films (Fig. 2b and Supplementary Section 1)^10^. For *T* < *T*_M_, we observe large OOP AFM domains (Fig. 2b, purple) separated by antiphase domain walls (ADWs) with IP AFM order (Fig. 2b, yellow/orange). As the system is warmed, the ADWs widen and small IP islands nucleate and progressively increase in size. At *T* ≈ *T*_M_, the ADW length scale diverges as the anisotropy approaches zero, resulting in a complex distribution of domains hosting nearly equal fractions of IP and OOP regions. At *T* > *T*_M_, IP regions enlarge and become dominant, whereas OOP regions shrink dramatically. Nonetheless, we still observe several OOP regions across the sample.

To determine the topological character of the AFM textures, we constructed Néel vector maps for *T* > *T*_M_. We used red–green–blue (R–G–B) colours to denote IP domains with spin directions at 120° from each other, as expected from the underlying trigonal symmetry^26^. On the basis of previous work in attached films^10^, we expect topological textures to be associated with small OOP ‘cores’. Although such small topological cores are usually difficult to detect with LH-polarized X-rays, we observed some larger OOP ‘bubbles’, not all of which are associated with a whirling texture and are therefore likely to be topologically trivial and produced by pinning of the OOP phase at local defects. More importantly, we were able to observe many topological AFM textures, including AFM merons and antimerons (Fig. 2c). Individual AFM (anti)merons can be characterized by an AFM winding number ±1 (depending on whether the texture whirls along or opposite to the azimuth angle), and an AFM topological charge ±1/2 (depending on the product of the winding number and core polarization)^4,9–11,26^. The complete 360° winding of these topological textures can be confirmed using Néel vector mapping (Fig. 2c, refs. ^10,26^ and Methods) or direct readout of the vorticity by mapping stray fields generated from the whirling canted magnetization using diamond magnetometry^11^. Moreover, (anti)merons can locally combine to form pairs, which may have a net AFM topological charge of ±1 (bimerons) or 0 (topologically trivial pairs), depending on the relative core polarization of (anti)merons. It should be noted that bimerons and topologically trivial pairs cannot be distinguished by XMLD techniques^10^. The observation of a multichiral topological AFM family unequivocally confirms that our membranes harbour the Kibble–Zurek phenomenology originally discovered in attached films^10^, despite the larger concentration of structural defects. Noteworthily, topological states are observed in the absence of any high spin–orbit heavy-metal overlayer, indicating that the creation and stabilization of topological order are intrinsic to α-Fe_2_O_3_ and do not rely on interfacial interactions present in typical FM skyrmionic systems^1,5,8,9^.

A clear difference between membranes and attached films is that AFM textures in the former are more strongly pinned than in the latter; therefore, texture patterns are reproduced almost identically even after performing multiple thermal cycles across *T*_M_ (Supplementary Section 2). We hypothesize that texture pinning results from highly localized alteration of the magnetic properties due to an increased density of point and extended defects in the membranes. This reasoning is supported by previous studies extensively performed in other topological systems^28,29^. We also performed in situ imaging with magnetic fields and found the AFM state to remain largely unperturbed (Supplementary Section 2), indicating that our topological textures are much more robust compared with counterparts observed in synthetic AFMs^5^.

## Flexure-driven state reconfiguration across three-dimensional folds

**Fig. 3:**
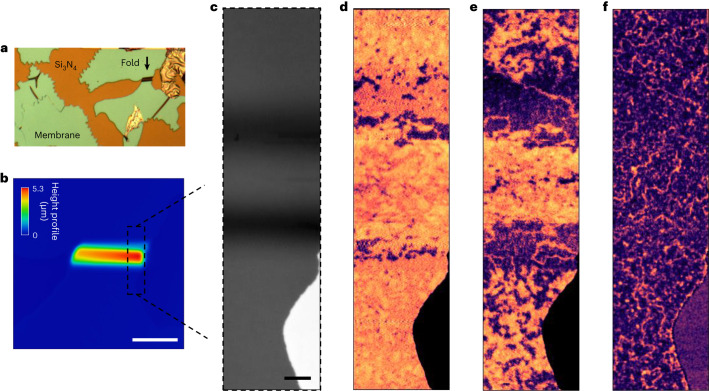
Flexure-driven spatial reconfiguration of AFM states. **a**, Optical microscopy image indicating the folded region being studied with a black arrow. **b**, Three-dimensional height profile map of the folded region in a buffered membrane (type C), shown from the top, obtained using confocal microscopy (Methods). The colour legend indicates the height profile. Scale bar, 20 µm. **c**, Fe L_3_-edge X-ray transmission contrast obtained at the right side of the folded membrane, as approximately indicated with a dashed box in **b**. Our characterization confirmed that the buffered membrane was oriented with the α-Fe_2_O_3_ side facing up and the buffer side facing down (Methods). Scale bar, 2 µm. **d**–**f**, AFM STXM contrast obtained across different temperatures: $T > {T}_{{\rm{M}}}^{\;{\rm{FF}}}$ (**d**), $T \approx {T}_{{\rm{M}}}^{\;{\rm{FF}}}$ (**e**) and $T < {T}_{{\rm{M}}}^{\;{\rm{FF}}}$ (**f**). The OOP and IP contrasts are indicated in purple and yellow/orange, respectively, as that in Fig. 2. The AFM textures across the folds were mapped out by ‘stitching’ together multiple images, and optimizing the focal point for each corresponding region.

One of the most remarkable properties of free-standing membranes is their extreme mechanical flexibility^16,17,22^, which could be used to tune strain-/structure-sensitive magnetic properties^30–32^. We find that our buffered α-Fe_2_O_3_ membranes are not brittle, as one expects from ceramic-like oxides, but are very flexible and can develop ‘folds’. An example is illustrated in Fig. 3, where the fold has a maximum curvature of ∼3 × 10^−4^ nm^−1^. In extreme scenarios, we even observe complete 180° ‘folded-over’ membranes (Supplementary Section 3). Large-area buffered membranes (type C) are particularly remarkable, as they can hold complex strain distributions without undergoing fracture.

To study the impact of flexure, we imaged naturally flexed regions across the membrane folds that serendipitously emerged on direct transfer. Their shapes were confirmed through confocal microscopy, which maps the membrane’s height profile (Fig. 3b and Methods). Moreover, the slopes of the flexed region appear darker in STXM images (Fig. 3c) because the signal exponentially diminishes with the effective sample thickness, *t*_eff_ ≈ *t*/cos*θ* (*t*, actual thickness; *θ*, deviation from the horizontal).

Flexure effects are immediately apparent from the images collected through the Morin transition. We define ${T}_{{\rm{M}}}^{\;{\rm{FF}}}$ as the Morin transition in the far-field (flat) region of the membrane away from the fold. The data in Fig. 3 were collected on a type-C membrane with the α-Fe_2_O_3_ layer facing upwards, and the buffer layer lying underneath (Methods). Above ${T}_{{\rm{M}}}^{\;{\rm{FF}}}$ (Fig. 3d), both far-field regions and peak of the fold exhibit an IP AFM matrix hosting several OOP regions, as expected. However, narrow bands near the base of the fold primarily host OOP AFM regions. On cooling to $T \approx {T}_{{\rm{M}}}^{\;{\rm{FF}}}$ (Fig. 3e), the far-field regions exhibit mixed IP and OOP contrast. The base of the fold has a robust OOP matrix with clear AFM ADWs, whereas the top of the fold remains in the IP state. Finally, at temperatures well below ${T}_{{\rm{M}}}^{\;{\rm{FF}}}$, all the regions are in the OOP state interspersed with ADWs (Fig. 3f). This remarkable evolution is consistent with the magnetic anisotropy (and hence the Morin temperature) changing rapidly through the fold so that ${T}_{{\rm{M}}}^{\;{\rm{peak}}} < {T}_{{\rm{M}}}^{\;{\rm{FF}}} < {T}_{{\rm{M}}}^{\;{\rm{base}}}$. This is in stark contrast with the behaviour in flat membranes, which are rather homogeneous. Qualitatively, our observations are consistent with Morin temperatures being raised near the base and lowered at the peak.

To further explore these effects, we imaged a type-C buffered membrane in the reversed configuration, with the buffer layer facing upwards and the α-Fe_2_O_3_ layer underneath (Supplementary Section 4). Here the fold has the opposite effect relative to the trend shown in Fig. 3: the OOP state is stabilized on top of the fold (consistent with an increase in *T*_M_), whereas the topologically rich IP state is stabilized at the base (decrease in *T*_M_). Hence, for these flipped membranes, we find that ${T}_{{\rm{M}}}^{\;{\rm{base}}} < {T}_{{\rm{M}}}^{\;{\rm{FF}}} < {T}_{{\rm{M}}}^{\;{\rm{peak}}}$. The importance of the buffer layer is further confirmed by measurements performed on unbuffered samples (type B; Supplementary Section 5), which do not display any magnetic-state change across folds. Finally, to confirm the presence of topological textures on folds, we also performed vector mapping and found a clear instance of a meron–antimeron pair near the base of the fold (Supplementary Section 6). These observations demonstrate that one can modulate the magnetic properties of α-Fe_2_O_3_ membranes on a scale of a few micrometres, controlling with precision the regions where topological textures can form.

## Strain and anisotropy modelling

One natural interpretation of our results is that the change in *T*_M_ is produced by flexure-induced strain. Magnetic anisotropy in α-Fe_2_O_3_ results from a delicate competition between dipolar and on-site interactions that are sensitive to structural variations^20,30,33^. In particular, epitaxial studies^30^ revealed that uniform compressive and tensile strains applied via substrate clamping raise and lower *T*_M_, respectively (Supplementary Section 8). Interpreting our results using this insight also highlights the role of the buffer layer since, at any given point on the fold, the thickness-average strain is expected to be zero to the first order for a homogeneous (unbuffered) membrane (see below).

**Fig. 4:**
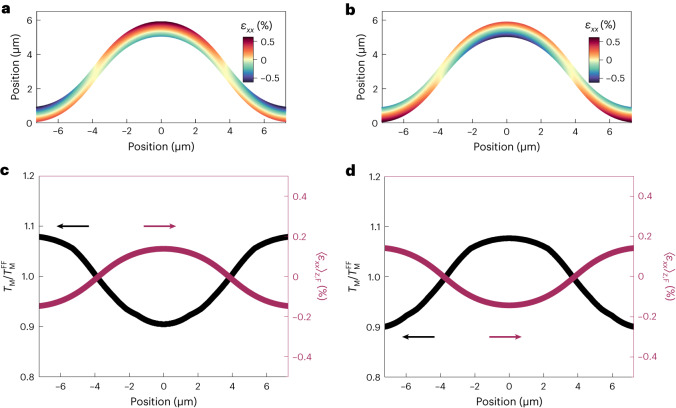
Flexure strain and anisotropy model. **a**,**b**, Non-uniform strain distribution, *ε*_*xx*_, in the α-Fe_2_O_3_ layer as a function of thickness and length, across folds in buffered AFM membranes (type C) with α-Fe_2_O_3_-side-facing-up (**a**) and buffer-side-facing-up (**b**) configurations. Membrane thickness has been exaggerated to aid the visualization of the non-uniform strain distribution as a function of thickness. The neutral line (*ε*_*xx*_ ≈ 0), indicated in yellow, is located at very different positions in **a** and **b** relative to the middle of the α-Fe_2_O_3_ layer, resulting in strong variations in the average and maximum strains in the AFM layer. **c**,**d**, Evolution of the thickness-averaged strain in the α-Fe_2_O_3_ layer, 〈*ε*_*xx*_〉_*z*,F_, and the corresponding local ${T}_{{\rm{M}}}\,/\,{T}_{{\rm{M}}}^{\;{\rm{FF}}}$ for the two configurations given in **a** (**c**) and **b** (**d**). Strain-driven modulation of the local *T*_M_ was obtained from the model developed in the literature^30^. Here ${T}_{{\rm{M}}}\,/\,{T}_{{\rm{M}}}^{\;{\rm{FF}}}$ larger and smaller than unity refers to the elevation and suppression of the local Morin temperature, respectively, and therefore the magnetic anisotropy, relative to the flat far-field regions.

To develop this simple insight further, we numerically calculated the strain distribution across our fold through a finite-element mechanical model of a buffered membrane (Methods), whose flexed region closely reproduces the profile determined by confocal microscopy. We find that flexure results in sizable uniaxial IP tensile and compressive strains, *ε*_*xx*_, distributed along the membrane thickness (*z* direction), such that the neutral (unstrained) line^16^ is located close to the middle of the buffered membrane. Due to the presence of the buffer, which itself accommodates some strain, the net strain averaged across the α-Fe_2_O_3_ layer 〈*ε*_*xx*_〉_*z*,F_ is actually non-zero. Moreover, the strength of 〈*ε*_*xx*_〉_*z*,F_ gradually varies across the length of the fold, changing sign near the point where the curvature is zero (Fig. 4a). Finally, this model predicts that reversing the buffered membrane should reverse the sign of strain distribution (Fig. 4b).

To place this observation on a more quantitative ground, we calculated the local *T*_M_ (Fig. 4c) by combining the thickness-averaged strain profile from our mechanical model with the strain dependence of *T*_M_ determined in the literature^30^. A caveat of this analysis is that the strains in other work^30^ were substrate induced and biaxial, whereas the flexure-induced strains here are primarily uniaxial. In buffered membranes with the α-Fe_2_O_3_ side facing up, we find that the net compressive strain at the base of the fold (${\langle {\varepsilon }_{xx}\rangle }_{z,{\rm{F}}}^{{\rm{base}}} < 0$) and the net tensile strain at the peak (${\langle {\varepsilon }_{xx}\rangle }_{z,{\rm{F}}}^{{\rm{peak}}} > 0$) should lead to an increase and decrease, respectively, in the local *T*_M_ by ∼10%. This model of the AFM-state reconfiguration is, in general, consistent with our experimental results determined from the STXM images taken across the fold (Supplementary Section 9). Furthermore, the sign of this effect is reversed for the flipped buffered membrane, whereas its magnitude remains approximately the same, consistent with our results (Supplementary Section 4).

Our model also explains the absence of any sizable modulation of *T*_M_ across the fold in unbuffered samples (Supplementary Section 5), since in this case, the neutral strain line lies approximately in the middle of the α-Fe_2_O_3_ layer so that ${\langle {\varepsilon }_{xx}\rangle }_{z,{\rm{F}}} \approx 0$. We conclude that our model effectively explains the AFM-state reconfiguration observed across the folded membranes.

## Non-thermal generation of topological textures via strain

**Fig. 5:**
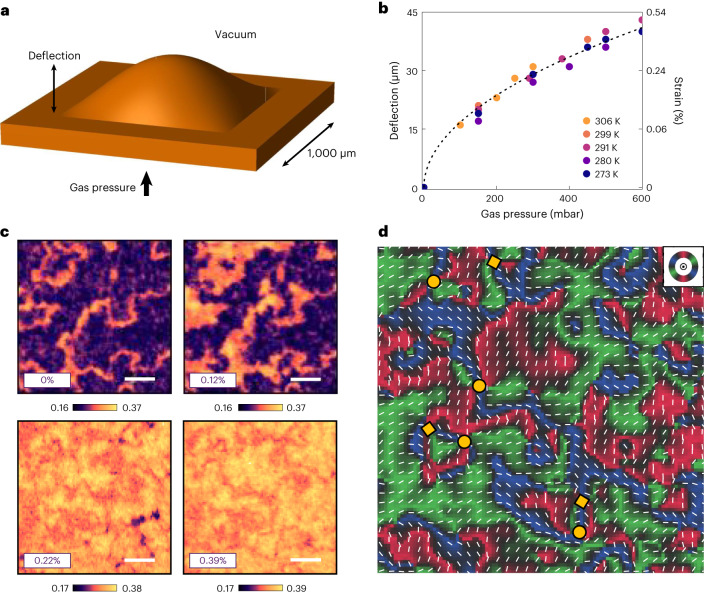
In situ strain tuning of AFM states and non-thermal generation of topological textures. **a**, Schematic of the α-Fe_2_O_3_ membrane strained in a controlled manner where the underlying flexible Si_3_N_4_ support was square in shape. The surrounding membrane lying on top of the rigid silicon frame remained flat. The deflection corresponds to the maximum vertical displacement of the centre of the membrane. Dimensions are not to scale. **b**, Pressure-dependent calibration of the deflection (left axis) and estimated IP tensile strain (right axis) performed across a range of temperatures. The dashed black curve corresponds to a square-root fit of deflection versus pressure, which is consistent with the trends reported in the literature^34^. **c**, Strain-dependent evolution of the AFM STXM contrast obtained at the Fe L_3_-edge whilst pressurizing the buffered membrane (type C) at room temperature (*T* < *T*_M_). The OOP and IP contrasts are indicated as purple and yellow/orange, respectively, as that in Fig. 2. Scale bars, 1 µm. All the images were recorded at the same position. The energy-contrast scale was slightly varied across the transition to aid visualization. **d**, Néel vector map performed at 0.39% strain in the same position as that in **c**. R–G–B colours (key inset in **d**) and thin white bars represent the IP AFM orientations. The yellow circles and squares indicate AFM merons and antimerons, respectively.

In the previous section, we illustrated how the magnetic anisotropy of α-Fe_2_O_3_ (and consequently the *T*_M_) can be effectively controlled by local strains over length scales of a few micrometres. This principle could be employed, for example, to localize the topologically rich phase in pools or channels by engineering appropriate strain patterns. A more immediate application of our findings is the demonstration that topological textures can be non-thermally created by traversing the spin-reorientation transition in the temperature–strain phase space at a constant temperature—effectively defining an isothermal analogue of the Kibble–Zurek transition. To this effect, we deployed a gas-cell manipulator (Fig. 5a,b and Methods). Here the variation in the internal gas pressure of the cell flexed the Si_3_N_4_ support, resulting in the controlled in situ straining of the AFM membrane. We investigated a flat membrane attached to a square-shaped support. Flexing this support generates biaxial strain, the value of which can be accurately determined by measuring the deflection of the membrane through the change in the focal position of the microscope^34^. It should be noted that the geometry of this configuration ensures that the strain is purely tensile at the centre of the square, regardless of the presence or position of the buffer layer^34^. At room temperature and in the absence of any strain, our sample was in the OOP state (*T* < *T*_M_; Fig. 5c). Pressurizing the cell resulted in a gradual enhancement in the IP domains, as the tensile strain suppresses the magnetic anisotropy (Supplementary Section 8)^30^. At higher gas pressures, the membrane transitioned into an IP state with a few small OOP patches, very similar to the state found at high temperature and zero strain. Finally, we reconstructed a Néel vector map in the strained membrane to reveal the local IP variation of the spin textures. Thus, we were able to confirm the presence of a family of topological AFM textures hosting non-trivial winding, including (anti)merons and bimerons (Fig. 5d). The entire imaging sequence was isothermally performed at room temperature. Hence, this in situ experiment evidences that strain tuning can be used to non-thermally recreate the Kibble–Zurek-like phenomenology, akin to the evolution we previously observed across the Morin transition (Fig. 2).

## Discussion and outlook

Using a powerful transmission-based AFM vector mapping technique, we demonstrate that free-standing α-Fe_2_O_3_ membranes host a family of IP and OOP AFM states, including textures that are evidenced to be topological. Our results suggest that the background AFM in which these textures occur can be modulated by flexure-induced strain in three-dimensional folded structures. Moreover, using an in situ strain manipulator, we showed that controlled structural tuning can be exploited to realize non-thermal Kibble–Zurek-like generation of topological AFM states at room temperature.

At the fundamental level, our results suggest that strain modulation has the potential to design and manipulate topological AFM textures, adding a completely new vista of magnetic topology to the burgeoning research landscape built on exploiting membranes of quantum materials to generate exotic states^16,17,22^. Our results also pave the way for the exploration of static and dynamical AFM evolution^35–38^ triggered by in situ electric, magnetic, optical or structural perturbations^9,11,39^. For example, we envisage the electrical triggering of topological reconfiguration and dynamics via localized piezoelectric control. Moreover, by integrating extremely flexible AFM membranes/ribbons onto carefully designed three-dimensional nanostructures, it may become possible to induce novel symmetry-breaking exchange or anisotropy interactions, for example, through curvilinear geometric^31,32^ and magnetoelastic^40–42^ effects^43^, thereby enabling the design of spatially varying magnetic states^42,44^, or the realization of hitherto undiscovered chiral textures^45–47^.

On the applied front, the development of substrate-free AFM membranes that preserve magneto-crystalline properties and topological order addresses a major roadblock inhibiting the integration of crystalline AFM materials into established spintronics platforms. Specifically, complex and dense topological AFM fabrics are expected to possess fast nonlinear dynamics^2,48^, which could open explorations into AFM-based silicon-compatible ultrafast reservoir computing^7,49^ or dense AFM memory-in-logic arrays in three dimensions^50^.

## Methods

### Membrane growth and fabrication

Throughout this work, we have studied Rh-doped α-Fe_2_O_3_ (α-Fe_1.97_Rh_0.03_O_3_) membranes. Rh doping was used to elevate the Morin transition temperature beyond what is typically achievable in undoped counterparts, as discussed in the literature^10,20^. Membrane layers were grown either on (111)-oriented STO or (001)-oriented α-Al_2_O_3_ substrates from CrysTec, using a pulsed laser deposition setup fitted with a KrF excimer laser. First, the growth of the water-soluble Sr_3_Al_2_O_6_ layer was performed at 950 °C, in a pure oxygen atmosphere of 1 mtorr, and a laser repetition rate of 2 Hz. The buffer SrTiO_3_ layer (thickness, ∼3 nm) was then deposited at 850 °C, 10 mtorr and 2 Hz. This was followed by the LAO layer (∼10 nm) grown at the same temperature and repetition rate, with an oxygen pressure of 1 mtorr. Subsequently, the α-Fe_2_O_3_ layer (∼30 nm) was deposited at 700 °C, 2 mtorr and 3 Hz. Finally, the samples were gradually cooled in a high-oxygen-pressure environment to minimize the oxygen vacancies formed during the growth. Each layer in the heterostructure was grown using a corresponding individual target.

We fabricated free-standing α-Fe_2_O_3_ membranes using the selective water-etching technique^15–17,21,22,51,52^. To delaminate the membranes, the samples were placed in high-purity deionized water at room temperature to dissolve the SAO layer. In the case of direct transfer (Fig. 1a), the membranes gradually floated to the top of the water surface after SAO dissolution, following which they were scooped out using the desired support (Si, Si_3_N_4_ and so on). The presence of non-uniform forces in the scooping process can result in the serendipitous formation of folded regions. These flexed geometrical structures are held in place due to van der Waals interactions with the underlying support. Alternatively, in the case of indirect transfer, a temporary support consisting of poly(methyl methacrylate) (PMMA) was spin coated on top of the sample, which was then held by a flexible tape. The entire stack was then placed in deionized water. After delamination, the membrane was carefully moved to the final support (Si, Si_3_N_4_ and so on) using a transfer stage that was held onto the tape. Last, the PMMA layer and tape were removed from the top surface of the membrane through a room-temperature acetone wash. The indirect process enables the targeted and controlled transfer of membranes with a much higher yield compared with its direct counterpart.

### Materials characterization

The structural quality and crystallinity of the samples were determined by XRD involving 2*θ*−*ω* scans, rocking curves (*ω* scan), *ϕ* scans and pole figure measurements (Fig. 1 and Supplementary Section 1). The measurements were performed for both as-grown films on crystalline substrates and membranes transferred onto Si substrates. The structural phase of α-Fe_2_O_3_ was further confirmed through Raman spectroscopy, performed using a Jobin Yvon Horiba LabRAM Evolution spectrometer in reflection geometry (514.5 nm laser). Transmission-electron-microscopy-based SAED experiments were carried out using a JEOL JEM-ARM200F instrument equipped with a cold field-emission gun, operated at 200 kV. To ensure electron transparency, the AFM membranes were mounted on commercial 30 nm Si_3_N_4_ holders fabricated on top of Si substrates from Agar Scientific. The magnetic characterization was performed using a Quantum Design magnetic property measurement system superconducting quantum interference device system on field-cooled samples under a 5,000 Oe field during warming and cooling measurement scans. The detached membranes were supported on Kapton tape for magnetometry. Supplementary Section 1 shows the Morin transitions of various membranes. For type-C membranes discussed in the main text, the transition occurs between 305 and 315 K. Although there is generally good correspondence between magnetometry and STXM imaging, in some cases, the temperature dependence may have a minor difference, most probably due to small strain variations introduced during the corresponding sample preparation steps and different thermal properties of the supporting layers. In Supplementary Section 12, by contrasting the thermal evolution of the STXM experiments against that of the superconducting quantum interference device magnetometry data, we find that an upward shift of ∼7.5 K is required to correct the STXM temperature estimate. The shape and height profiles of the membrane folds were studied by confocal microscopy using the Sensofar S neox metrology tool.

### STXM imaging

Fe L_3_-edge resonant STXM imaging was performed at the PolLux X07DA endstation of the Swiss Light Source^53^. The images were obtained by recording the transmission of normally incident X-rays, polarized either linearly (XMLD-STXM) or circularly (XMCD-STXM), and focused using a Fresnel zone plate (FZP). The outermost zone width of the FZP was selected, in tandem with the size of the monochromator exit slits of the beamline, resulting in a spatial resolution of about 40–50 nm. As the focusing efficiency of a diffractive optical element is not unitary, an order-selecting aperture, combined with a centre stop fabricated on the FZP, was employed to guarantee that only the focused X-rays illuminate the sample. The FZP and order-selecting aperture are indicated in Fig. 2a. An image was then obtained by scanning the sample with a piezoelectric scanner and recording the transmitted X-ray intensity for each point in the image. Typically, the field of view of our images had a square or rectangular shape.

For X-ray transmission experiments, the AFM membranes were mounted on commercial 100 nm Si_3_N_4_ holders fabricated on top of Si substrates from Silson. The membrane temperature was controlled using a thin Au/Ti heater coil, lithographically fabricated directly onto the Si_3_N_4_/Si holders. Passing a current through the heater coil leads to resistive dissipation, thereby heating the sample. The resistance of the heater is calibrated and can be simultaneously measured to monitor the sample temperature. Supplementary Sections 3 and 4 show these Au/Ti heaters. For the in situ field studies, magnetic fields were applied in the plane of the samples (that is, in the crystal basal planes) using a rotatable permanent-magnet setup, which could apply fields up to ∼120 mT (Supplementary Section 2).

XMLD-STXM imaging was performed by collecting a pair of images, using LH X-ray polarization at two photon energies near the Fe L_3_-edge (around ∼710 eV), namely, *E*_1_ and *E*_2_, chosen to provide the maximum AFM contrast in our samples. The XMLD energy contrast was then calculated as $\varDelta =({I}_{{E}_{1},{\rm{LH}}}-{I}_{{E}_{2},{\rm{LH}}})/({I}_{{E}_{1},{\rm{LH}}}+{I}_{{E}_{2},{\rm{LH}}})$. On the basis of crystal symmetry analysis^10^, it can be shown that the LH XMLD intensity varies as *I* = *I*_A_ + *I*_B_cos^2^*ψ*, where *ψ* is the relative angle between the linearly polarized X-ray electric field and magnetization, allowing us to map out the local AFM order. This immediately reveals that XMLD imaging is unable to distinguish IP AFM orientations separated by 180°. Likewise, it is also not possible to resolve the direction of OOP AFM orientation^10,26^. The absolute value of the XMLD energy contrast can depend on the beamline setup, detector and sample holder conditions. As the predominant AFM textures vary with temperature and strain, the intensity range from the energy contrast also changes. Hence, we have used the same colour scheme (purple to yellow/orange for OOP to IP) with different energy-contrast limits to aid visualization across the transition^10^. Note that this does not affect any of our scientific conclusions, as the IP-domain versus OOP-domain orientations can be unambiguously distinguished based on the angle-dependent data. Whilst performing imaging across curved membranes, we ensured that the X-ray polarization was always perpendicular to the direction of the fold, allowing the XMLD-STXM contrast in our images to be consistent across the fold, irrespective of the curvature. Next, the XMCD-STXM image presented in Supplementary Section 1 was acquired by taking a dataset with both right- and left-circularly polarized X-rays at a fixed energy *E*_*i*_. The XMCD contrast was calculated as $\delta =({I}_{{E}_{i},{\rm{RCP}}}-{I}_{{E}_{i},{\rm{LCP}}})/({I}_{{E}_{i},{\rm{RCP}}}+{I}_{{E}_{i},{\rm{LCP}}})$. Owing to the absence of strong FM textures in the sample, XMCD-STXM images showed negligible contrast. The data reduction was performed using a custom-built MATLAB tool (available via the public repository at https://gitlab.psi.ch/microspectro-public/).

Spatially averaged X-ray absorption spectra were obtained at the Fe L-edges from the transmitted signal by scanning a straight line that straddled across regions both on and off the membrane (Supplementary Section 1). The signal measured outside the membrane was used as the reference signal for the normalization of the spectra acquired on α-Fe_2_O_3_.

The depth of focus of the FZP utilized for the experiments reported in this work is ∼1 µm, meaning that the imaging of the folded membranes had to be performed in several steps, bringing different parts of the fold into focus. Finally, the composite images of AFM textures across the folds were produced by ‘stitching’ together multiple images.

To study the effects of flexure-induced strain in buffered membranes, it was crucial to determine whether α-Fe_2_O_3_ was on top or bottom of the stack, as non-uniform forces introduced during the scooping process can flip the membrane. To make this determination, we performed depth-sensitive local elemental mapping, which can be accomplished by either of the two following techniques: (1) energy-dispersive spectroscopy in transmission electron microscopy or (2) STXM imaging performed in the total electron yield detection geometry (Supplementary Section 3). The latter was performed using a channeltron detector biased to a voltage of 2.4 kV. Here only the secondary electrons emitted by the first few monolayers at the surface of the membrane are detected, in contrast to a typical transmission measurement where the entire thickness of the sample is probed. This allowed us to determine the orientation of the membrane, depending on whether chemical contrast was detected at the Fe L_3_-edge or La M_5_-edge (Supplementary Section 3).

### Néel vector maps

The IP Néel vector maps (Figs. 2c and 5d and Supplementary Section 6), which provide orientational information of the IP AFM order, were constructed by combining the energy-contrast XMLD-STXM images obtained at six azimuthal sample rotation angles about the crystallographic *c* axis in the range of −45° to +45°, where the limits were set by the in situ rotation stage (PolLux beamline; Fig. 2a). For gas-cell vector map studies, the sample remained fixed as the X-ray linear polarization was varied in the range of 0° to 90° (SIM beamline). Supplementary Section 11 provides further details. Theoretical and experimental details supporting this approach to Néel vector reconstruction can be found in our previous studies^10,26,27^. For each pixel in the field of view, we fit the angular dependence of the XMLD (discussed earlier) to extract the average spin direction. Owing to the trigonal symmetry and weak basal-plane anisotropy of α-Fe_2_O_3_, we mapped the spin directions using the R–G–B colour scale, which indicates the IP directions of the AFM order. For easy identification, the local IP Néel vector direction is indicated using a thin white bar. Regions in these maps where the AFM axis was identified to be OOP were coloured white^10^. Néel vector maps were used for identifying the position of topologically non-trivial AFM textures (merons and antimerons), where the IP AFM order undergoes a full ±360° winding around a core, similar to our approach in other work^10,26^. We identify several topological textures, demarcated as yellow circles (merons) or squares (antimerons) (Figs. 2c and 5d and Supplementary Section 6), where we observe the complete winding or anti-winding of the IP AFM order around the core, that is, R–G*–B–R*–G–B* or R–B*–G–R*–B–G*, respectively (asterisks denote time inversion).

### Gas-cell strain manipulator

To systematically apply and tune the strain on α-Fe_2_O_3_ membranes in situ (Fig. 5), we utilized a custom-built gas-cell manipulator that can be integrated with the PSI beamlines. Exhaustive details on the construction of the setup can be found elsewhere^34,54,55^. In summary, the gas cell consists of a stainless steel and aluminium chamber, sealed on one side by a sample membrane placed on a Si_3_N_4_ sample holder (see the ‘Membrane growth and fabrication’ section) and on the other side by a separate X-ray-transparent empty Si_3_N_4_ membrane. The cell was placed in the STXM chamber under a vacuum and the internal pressure of the cell was varied by flowing He gas via a proportional–integral–derivative-controlled needle valve. The flow rate was tuned until a stable equilibrium cell pressure was obtained in the range of 0–600 mbar, which was used to hold the membrane at constant flexure and strain during STXM imaging. The Si_3_N_4_ windows used here were 50 nm thick and did not have any Au/Ti heaters. To perform XMLD vector mapping, we rotated the linear polarization of the incident X-ray beam from the undulator source. The sample in the gas-cell setup was kept fixed. The flexure of the sample was determined in situ based on the displacement of the sample *z* position required to bring the STXM image in focus, relative to its original position in the flat unpressurized configuration. The strain as a function of pressure was then calibrated using the model developed previously^34^, as per the following equation:

$${\varepsilon }_{{\rm{r}}}=\frac{2{h}^{2}}{3{r}^{2}},$$

where *ε*_r_ is the radial component of the strain tensor, *h* is the deflection height at the centre of the membrane and *r* is the radius of the membrane. This model exactly applies only for circular membranes but should be approximately valid close to the centre of the square membrane; hence, all the measurements were conducted within ~50 µm of the membrane centre.

### Modelling and simulations

A time-dependent finite-element analysis was carried out to quantitatively study the strain profile of folded membranes. The simulated composite stack consisted of both buffered membrane (30 nm α-Fe_2_O_3_/10 nm LAO) and 100-nm-thick Si_3_N_4_ support. Young’s moduli and Poisson’s ratios of the materials used in the simulation are based on the values reported in the literature^56–58^. The simulation starts with the composite stack lying flat at rest, to which an impulsive upward external force is applied in the middle of the membrane, causing a fold to emerge. Right after the removal of the external force, the two ends of the folded membrane are rigidly fixed to the Si_3_N_4_ support, with their separation distance corresponding to the experimental results from confocal microscopy (Fig. 3b). Finally, both membrane and support deform and relax to their respective equilibrium states within ∼1 µs simulation time. The resulting final state was found to closely reproduce the equilibrium state of a folded membrane held on the Si_3_N_4_ support. The only tuning parameter in the simulation is the length of the suspended composite membrane at rest, which is determined by matching the height of the folded membrane in the simulation to that in the experiment. Supplementary Section 7 provides further details on the spatial distribution of all the relevant strain tensor elements across the folded membranes.

The SAED patterns were simulated by implementing the description of interfering diffraction patterns from two overlapping lattices, as developed elsewhere^24,25^. The spatial moiré pattern is obtained as the sum of the two lattice functions, and the diffraction pattern is the Fourier transform of this combined function. The IP lattice parameters were chosen to correspond to experimental values of about 5.10 Å for α-Fe_2_O_3_ and 5.51 Å for LAO. For simplicity, we did not include the ultrathin STO layer in this simulation as it is much thinner than LAO in our buffered membranes.

## Online content

Any methods, additional references, Nature Portfolio reporting summaries, source data, extended data, supplementary information, acknowledgements, peer review information; details of author contributions and competing interests; and statements of data and code availability are available at 10.1038/s41563-024-01806-2.

### Supplementary information

Supplementary Information Supplementary Sections 1–12 and references.

## Data Availability

The data supporting the findings of this study are available within the Article and can also be accessed on the Oxford University Research Archive (10.5287/ora-4jrwznxdx). The STXM data reduction was performed using a custom-built MATLAB tool (https://gitlab.psi.ch/microspectro-public/).
