# Supplementary material for: Spatially reconfigurable antiferromagnetic states in topologically rich free-standing nanomembranes
Source: Nat Mater. 2024 Feb 19;23(5):619–26. doi: 10.1038/s41563-024-01806-2 (PMC11068574; doi:10.1038/s41563-024-01806-2)
Supplement: Supplementary file 1 — Supplementary Sections 1–12 and references. [file 41563_2024_1806_MOESM1_ESM.pdf]

nature materials

Article

https://doi.org/10.1038/s41563-024-01806-2

Spatially reconfigurable antiferromagnetic states in topologically rich free-standing nanomembranes

In the format provided by the
authors and unedited

Contents

S1: Structural and magnetic characterisation of membranes....................2
S2: Robustness of AFM states....................5
S3: Extreme membrane folds and their surface characterisation....................6
S4: Flexure effects on AFM textures in a flipped membrane....................7
S5: Suppression of flexure effects in unbuffered membranes....................8
S6: Topological textures and IP domain reconfiguration at folds....................9
S7: Strain simulations....................10
S8: Dependence of the Morin transition on strain....................11
S9: Comparison of experimental and modelled AFM state distribution....................12
S10: Structural defects and moiré pattern in AFM membranes....................13
S11: AFM image processing steps....................14
S12: Temperature correlation....................15
References....................16

S1: Structural and magnetic characterisation of membranes

We investigated the crystallinity of various free-standing AFM membranes (types-A, B, C) by using selected area electron diffraction (SAED) in a transmission electron microscope, see Supplementary Figure S1.1a,c,e. Type-A, B membranes are unbuffered α-Fe_2O_3 layers grown on SAO-coated α-Al_2O_3 and $SrTiO_3$ substrates, respectively, whereas type-C membranes are buffered α-Fe_2O_3 layers grown on SAO-coated $SrTiO_3$ substrates. The buffer is made of LAO and STO layers (see Methods for details). SAED patterns confirm that the AFM layer in the type-A membrane is polycrystalline, whereas that in the type-B membrane is a single crystal. Buffered membranes of type-C are not only crystalline but also host a moiré pattern due to the mismatch with the LAO layer in the buffer. Furthermore, the epitaxial growth of various layers in buffered membranes is confirmed through pole figure analysis and ϕ-scans, illustrated in Supplementary Figure S1.2. Finally, optical microscopy images, shown in Supplementary Figure S1.1b,d,f, reveal that unbuffered type-A, B membranes usually host more cracks resulting in smaller intact membrane areas. By contrast, buffered type-C membranes usually form larger area samples with fewer cracks, which is important for realising strongly curved AFM structures to explore magneto-structural effects.

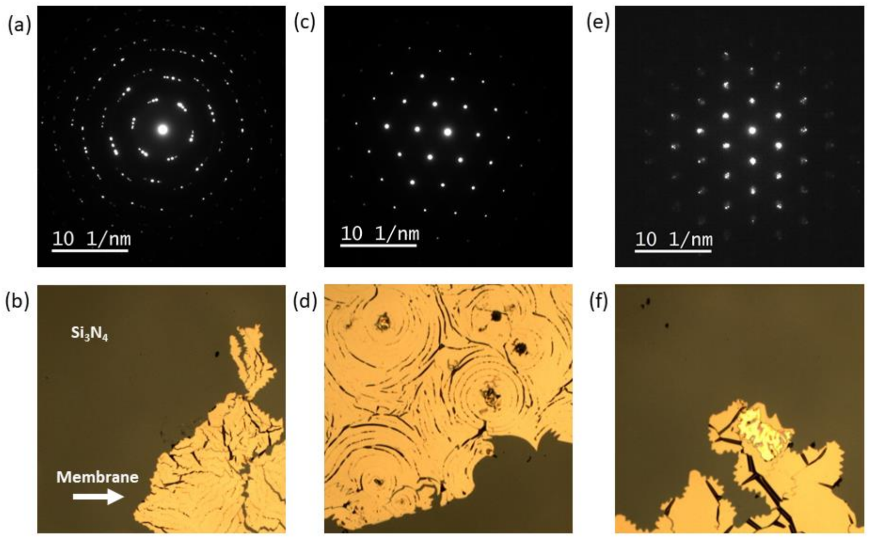

Supplementary Figure S1.1: SAED and optical characterization of unbuffered AFM membranes grown on **(a,b)** α-Al_2O_3 (type-A) and **(c,d)** STO (type-B), and **(e,f)** buffered membranes grown on STO (type-C). Details on the growth and transfer process are given in Methods and Figure 1. The zoomed SAED image of the type-C membrane clearly shows the satellite peaks due to lattice mismatch moiré in main text Figure 1e. The field of view in the optical images is 0.5 mm.

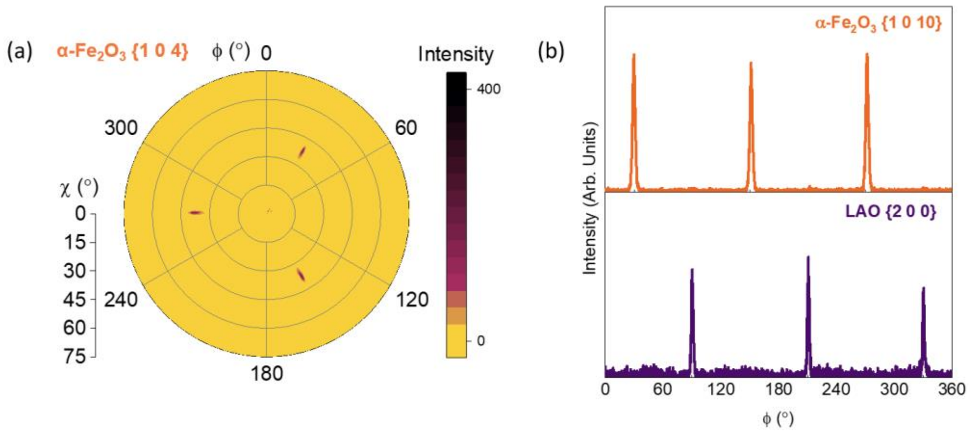

Supplementary Figure S1.2: Epitaxial growth of buffered membranes. (a) Pole figure showing the angular distribution of the α-Fe_2O_3 {1 0 4} Bragg peaks. **(b)** ϕ-scans of the α-Fe_2O_3 {1 0 10} and the LAO {2 0 0} Bragg peaks reveal a 30° offset between the corresponding in-plane orientations.

Next, temperature-dependent magnetometry reveals the hysteretic appearance and disappearance of the canted moment,[S1,S2] originating from bulk Dzyaloshinskii-Moriya interaction,[S3,S4] confirming the presence of a spin reorientation transition in all three types of freestanding membranes, see Supplementary Figure S1.3 and Methods. Membranes of type-A, C have T_M just above 300 K, whereas those of type-B have T_M slightly below 300 K. Despite the differences in crystallinity and T_M, all three types of freestanding AFM membranes generally exhibit similar phenomenology and AFM textures in scanning transmission X-ray microscopy (STXM), as evinced by main text Figure 2 and Supplementary Figure S1.4.

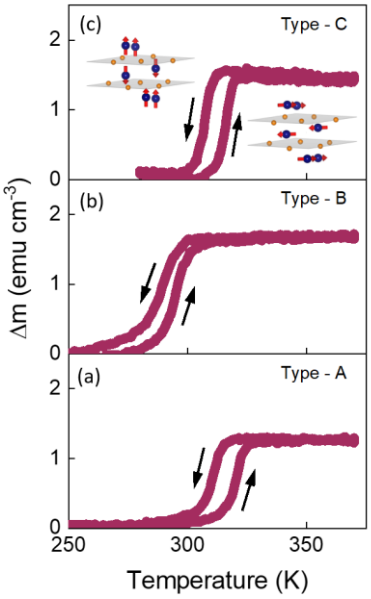

Supplementary Figure S1.3: Temperature-dependent magnetometry showing the change in the magnetisation of (a) type-A, (b) type-B and (c) type-C AFM membranes across the Morin transition emerging from the canted magnetism. Insets in (c) illustrate the layered AFM spin distribution and anisotropy in a sub-unit cell of α-Fe_2O_3 below T_M (out-of-plane configuration) and above T_M (in-plane configuration).

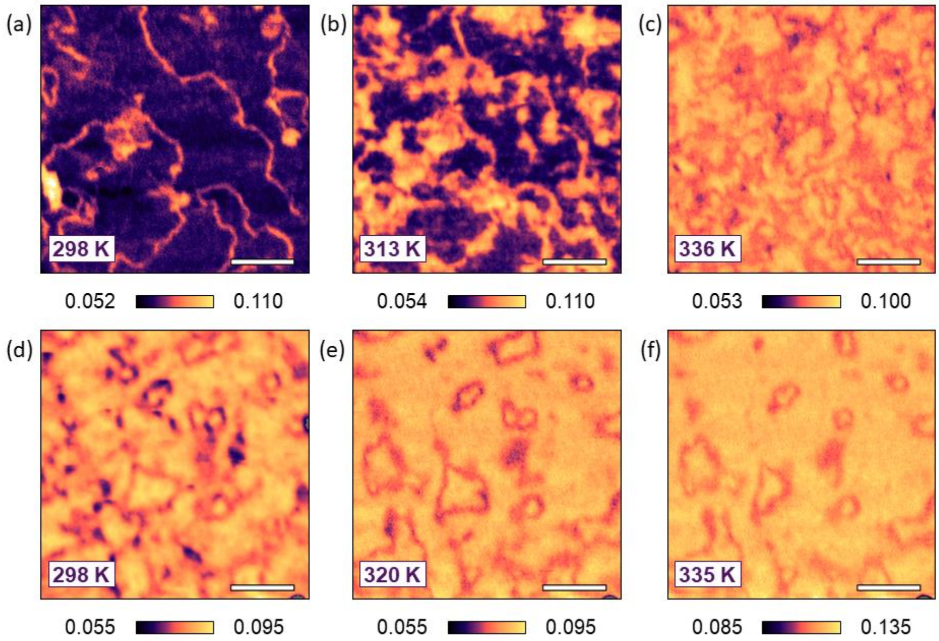

Supplementary Figure S1.4: Morin transition and AFM textures in type-A and type-B α-Fe_2O_3 membranes. Evolution of the AFM STXM contrast upon warming above room temperature, obtained at the Fe L_3-edge in unbuffered α-Fe_2O_3 membranes of **(a-c)** type-A and **(d-f)** type-B. The OOP and IP contrasts are indicated in purple and yellow/orange, respectively. The spatial scale bars are 2 μm long. Whilst the type-A membrane exhibits a clear transition upon warming above room temperature, its type-B counterpart does not, because the latter has a lower T_M, see Supplementary Figure S1.3.

We have also performed STXM imaging with circularly polarised X-rays (XMCD-mode) on our membranes (see Methods), which showed negligible contrast in comparison to imaging performed with linearly polarised X-rays (XMLD-mode), as illustrated in Supplementary Figure S1.5. This confirms that the observed magnetic order is primarily AFM, akin to previous work in attached epitaxial films.[S1]

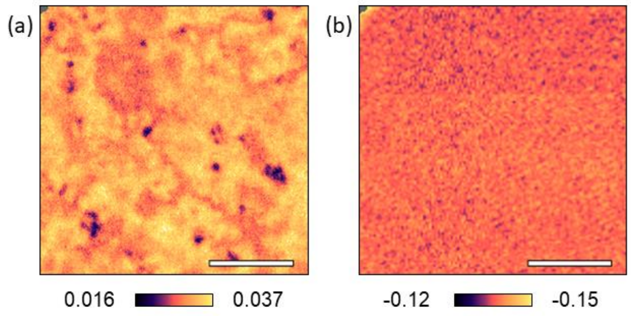

Supplementary Figure S1.5: Antiferromagnetic origin of the contrast. (a) XMLD-STXM and (b) XMCD-STXM obtained with linear and circularly polarised X-rays at the Fe L_3-edge in the topologically rich state, at $T > T_M$. The spatial scale bars are 2 μm long.

Lastly, the orientation of the AFM order, i.e. easy-plane vs easy-axis orientation in α-Fe_2O_3 can be determined via linear dichroic X-ray absorption spectroscopy (XAS), see Supplementary Figure S1.6. This is because the relative heights of the Fe L_2-edge peaks can be used to identify the AFM spin configuration.[S1,S2,S5] These XAS trends are consistent with the magnetometry results shown in Supplementary Figure S1.3 and STXM results in main text Figure 2.

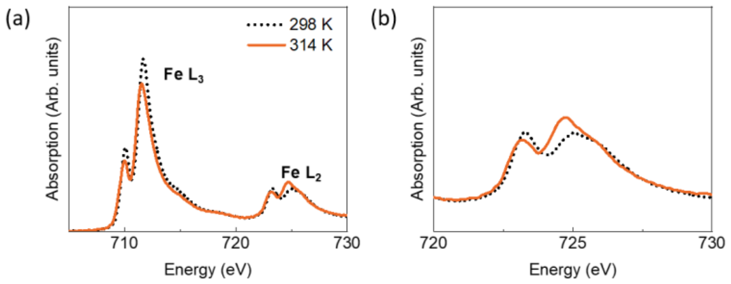

Supplementary Figure S1.6: X-ray absorption spectra and linear dichroism (a) revealed using normal-incidence LH-polarized X-rays (see Methods). **(b)** Zoomed-in view of the LH-XAS spectra shown in (a), near the Fe L_2-edge peaks clearly shows the reversal of the relative peak intensity, thereby, confirming the occurrence of the spin reorientation transition in α-Fe_2O_3.[S5]

S2: Robustness of AFM states

One of the key benefits of harnessing topological states in antiferromagnets is their robustness against external perturbations. We find that the AFM state remains largely unperturbed against *in situ* magnetic fields, see Supplementary Figure S2.1. This confirms that, despite being a canted AFM, α-Fe_2O_3 hosts textures that are more robust compared to those in synthetic AFMs.[S6]

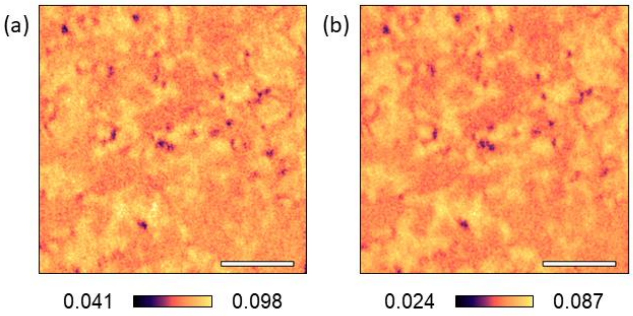

Supplementary Figure S2.1: Robustness of AFM textures against *in situ* magnetic field. (a,b) AFM STXM contrast from a buffered membrane (type-C) obtained in the topologically rich IP AFM state $T > T_M$, at (a) 0 mT and (b) 120 mT *in situ* IP magnetic field. The spatial scale bars are 2 μm long.

Furthermore, we also performed repeated Kibble-Zurek transitions by cooling and re-warming the membrane through the Morin transition, Supplementary Figure S2.2. In each instance we observe a complete transition to OOP and topologically-rich IP AFM states, respectively, akin to what is observed in main text Figure 2. Despite such a drastic change, the IP texture patterns are reproduced almost identically, presumably due to texture pinning at localised defects, as discussed in the main text.

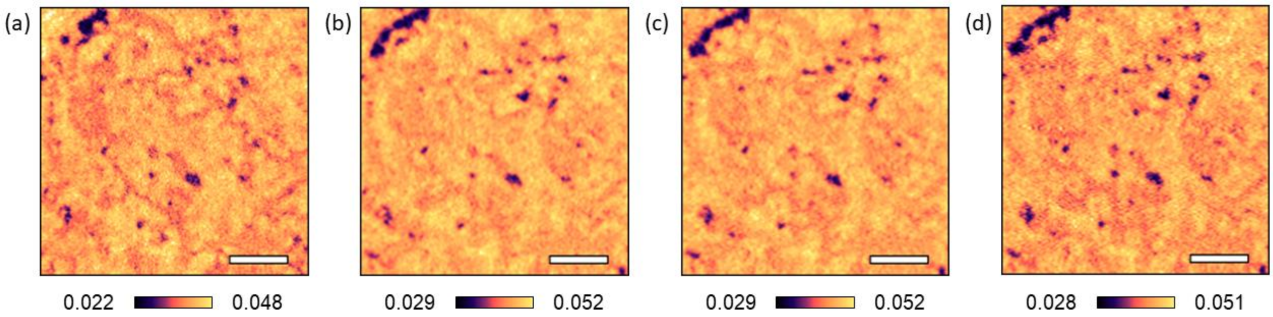

Supplementary Figure S2.2: Strong AFM texture pinning in the topologically rich IP AFM state, at $T > T_M$. The same texture pattern is reproduced even after repeatedly cycling the buffered AFM membrane (type-C) through the magnetic transition between 298 K and 314 K. **(a-d)** AFM textures observed after the (a) 1st warming step, (b) 2nd warming step, (c) 3rd warming step and (d) 10th warming step. The OOP and IP contrasts are indicated in purple and yellow/orange, respectively. The spatial scale bars are 2 μm long. All images were recorded at the same position.

S3: Extreme membrane folds and their surface characterisation

Flexed regions are often observed in membrane samples that are directly scooped from water, as illustrated in main text Figure 3, and Supplementary Figures S3.1, S4.1, S5.1. Whilst type-C membranes are ideal for observing such 3D structures owing to fewer cracks and larger area membranes, we also find instances of folds in type-B membranes. In extreme cases, we also observe membranes/ribbons that are completely 'folded-over', as shown in Supplementary Figure S3.1a. This is remarkable because oxides are typically inflexible and brittle in bulk form, yet they become highly flexible and elastic when grown as free-standing membrane crystals, opening pathways to experimentally explore curvilinear antiferromagnetism.[S7,S8]

We use this completely folded-over region to demonstrate our surface characterization technique, exploiting element-specific total electron yield (TEY) detection in the STXM setup (see Methods). This is useful for identifying the upper-most surface layer of buffered membranes (type-C), by imaging at the Fe L_3-edge and La M_5-edge, yielding maximal TEY signals when the α-Fe_2O_3-side and the buffer-side are facing up, respectively (see Supplementary Figure S3.1b-f).

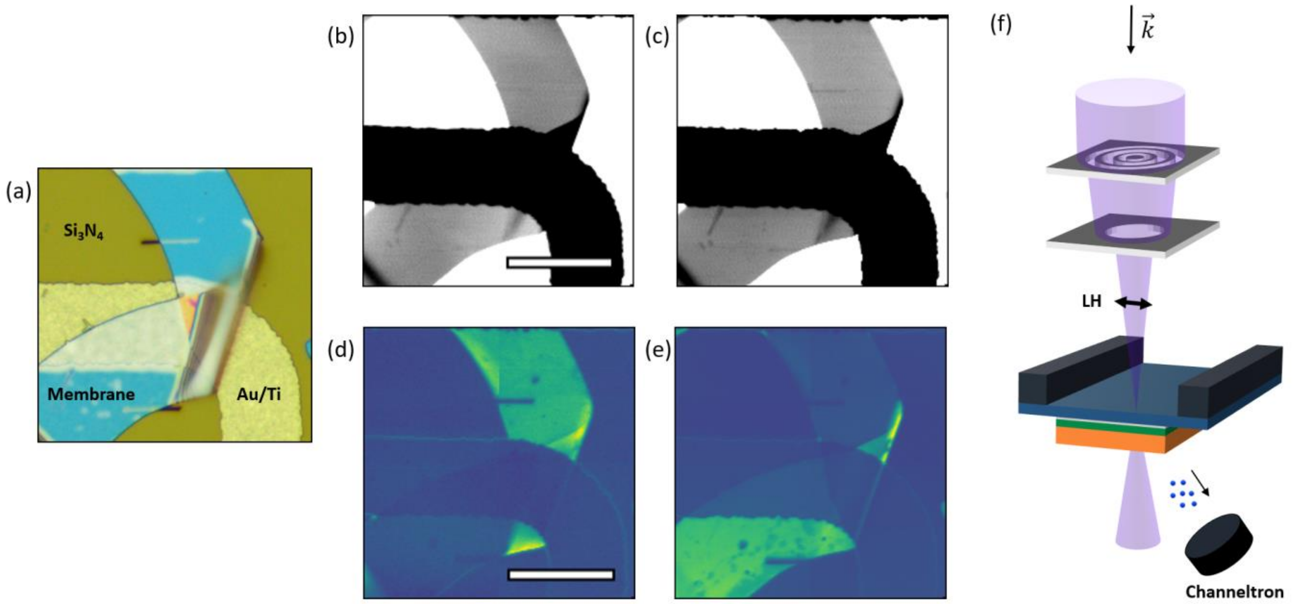

Supplementary Figure S3.1: Extreme flexibility of freestanding AFM membrane ribbons and their surface characterization. (a) Optical microscopy image reveals a 180° fold in a buffered AFM ribbon (type-C). **(b,c)** X-ray transmission images collected at the (b) Fe L_3-edge and (c) La M_5-edge show nearly uniform bulk-sensitive transmission across the buffered ribbon except in the regions of strong spatial curvature. X-ray transparent uncovered Si_3N_4 support appears white, whereas the X-ray opaque Au/Ti heater ring appears black. The spatial scale bars are 20 µm long. **(d,e)** TEY - STXM images collected at the (d) Fe L_3-edge and (e) La M_5-edge reveal surface-sensitive chemical information, in the same region. Images (d) and (e) confirm that the α-Fe_2O_3-side and the buffer-side of the ribbon, respectively, are facing up across the two sides of a completely folded-over membrane. The contrast is lower in the regions above the Au/Ti heater, due to the signal attenuation by the heater. Surfaces containing elements not present at the relevant X-ray edges appear dark in TEY images. The dark horizontal lines arise from carbon deposition during the X-ray spectroscopy scans; they are much more visible in TEY due to the surface sensitivity of the technique. **(f)** The geometry of surface-sensitive X-ray imaging performed in the TEY - mode in the STXM setup. Note that the sample is flipped compared to traditional bulk-sensitive X-ray transmission imaging shown in main text Figure 2. The channeltron detector is sensitive to photo-electrons emitted from the surface.

S4: Flexure effects on AFM textures in a flipped membrane

To test the validity of our interpretation of flexure effects we investigated buffered membranes that are ‘flipped’ such that the buffer-side is facing upward, and the α-Fe_2O_3-side facing down. As per the mechanical model in main text Figure 4, the average strain across the α-Fe_2O_3 layer should then also be reversed. This leads to a net compressive strain at the peak of the fold and the net tensile strain at the base, thereby, increasing and decreasing the local T_{M}, respectively. This is confirmed by our studies of a flipped membrane, illustrated in Supplementary Figures S4.1, S6.1 where the thermal evolution is opposite to that in main text Figure 3.

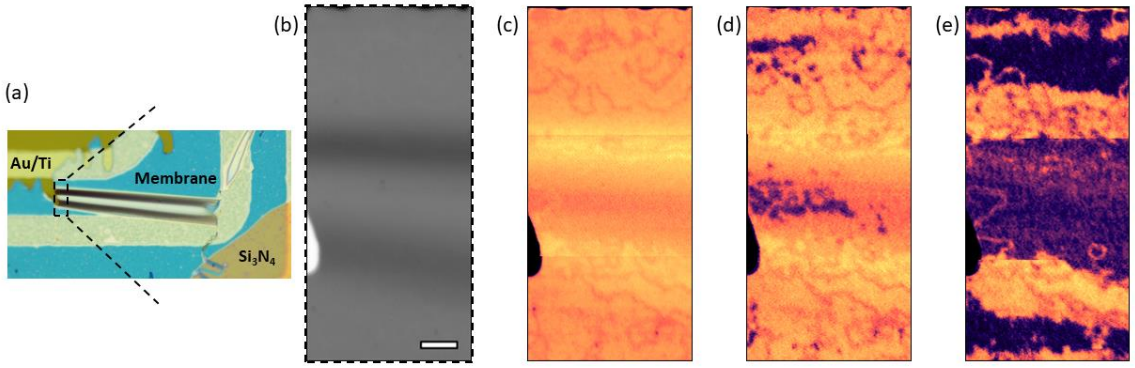

Supplementary Figure S4.1: Flexure-driven AFM reconfiguration in a flipped membrane. (a) Optical microscopy image indicating the folded region being studied. **(b)** Fe L_3-edge X-ray transmission contrast obtained using STXM at the left side of the folded membrane as shown in (b). The spatial scale bar is 2 µm long. Surface characterization experiments (see Methods and Supplementary Figure S3.1) confirmed that the buffered membrane was flipped with respect to main text Figure 3. **(c,d,e)** AFM STXM contrast obtained across different temperatures, (c) $T > T_{\mathrm{M}}^{\mathrm{FF}}$, (d) $T \sim T_{\mathrm{M}}^{\mathrm{FF}}$, and (e) $T < T_{\mathrm{M}}^{\mathrm{FF}}$. The OOP and IP contrasts are indicated in purple and yellow/orange, respectively.

S5: Suppression of flexure effects in unbuffered membranes

One consequence of our magneto-structural model is that the flexure effects should be markedly suppressed in unbuffered membranes (type-B), since the neutral line in a folded membrane falls approximately in the middle of the α-Fe_2O_3 layer, causing $\langle \varepsilon_{xx} \rangle_{z,\mathrm{F}} \sim 0$. This is confirmed by our experimental results in Supplementary Figure S5.1, suggesting that our mechanical model effectively explains the structural modulation of AFM properties.

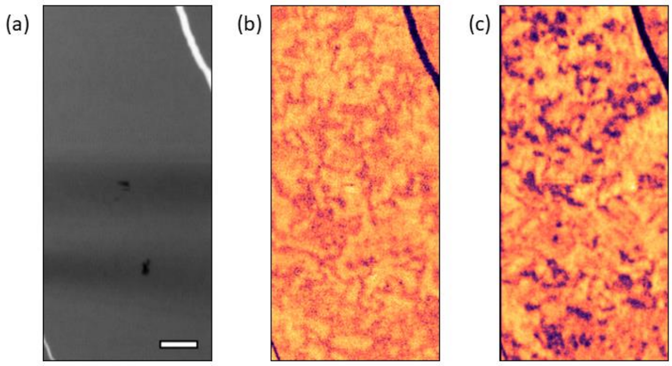

Supplementary Figure S5.1: Suppressed flexure-driven reconfiguration in unbuffered AFM membrane. (a) Fe L_3-edge X-ray transmission contrast obtained using STXM in an unbuffered membrane (type-B). **(b,c)** AFM STXM contrast obtained at (b) $T > T_{\mathrm{M}}^{\mathrm{FF}}$ and (c) $T \sim T_{\mathrm{M}}^{\mathrm{FF}}$, see Supplementary Figure S1.4. The spatial scale bar is 2 µm long. Spatial AFM reconfiguration resulting from flexure is observed to be weak, in stark contrast with the behaviour seen in buffered counterparts (type-C), shown in main text Figure 3 and Supplementary Figures S4.1, S6.1. It should be noted that while flexure effects are markedly suppressed in unbuffered membranes, they will not be identically zero due to the slight inherent vertical asymmetry of the AFM film grown epitaxially on a substrate with a different lattice constant.

S6: Topological textures and IP domain reconfiguration at folds

To confirm the presence of topological textures across membrane folds, we performed angle-dependent Néel vector mapping. As shown in Supplementary Figure S6.1, we observed an AFM meron-antimeron pair at the base of a fold, proving that the topological phenomenology expected across the Kibble-Zurek transition,[S1] see main text Figure 2, is also reproduced across strained AFM membranes. Hence, by reconfiguring the local AFM anisotropy, membrane flexure could also enable completely new forms of spatial control over AFM topology.[S9,S10,S11,S12]

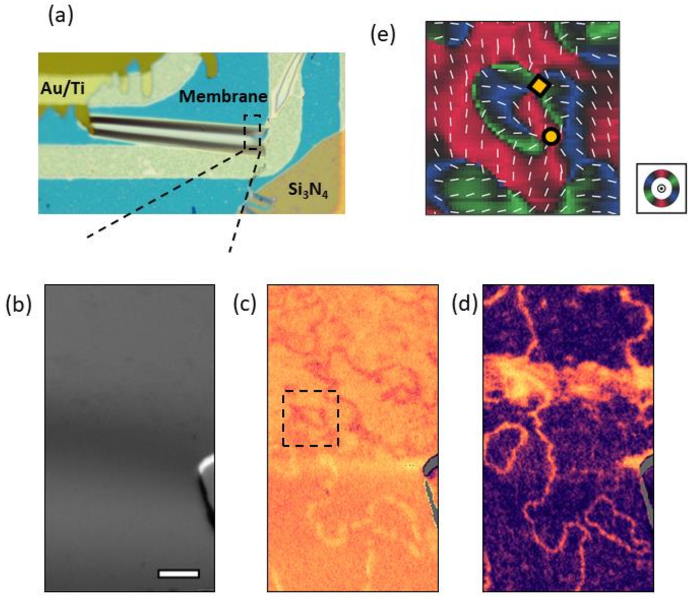

Supplementary Figure S6.1: Evidence of topological textures near membrane folds. (a) Optical microscopy image indicating the folded region being studied. **(b)** Fe L_3-edge X-ray transmission contrast obtained using STXM, in a buffered membrane with the buffer-side facing up. The spatial scale bar is 2 μm long. **(c,d)** AFM STXM contrast obtained at (c) $T > T_{\mathrm{M}}^{\mathrm{FF}}$ and (d) $T < T_{\mathrm{M}}^{\mathrm{FF}}$. **(e)** Vector-mapped LH-STXM image of the region indicated in (c), produced by rotating the sample azimuth (see Methods). R-G-B colours and thin white bars indicate IP orientations.

Vector map of the IP AFM state ($T > T_{\mathrm{M}}^{\mathrm{FF}}$) across the fold in Supplementary Figure S6.2 shows that the flexural strains can also reconfigure local IP domain sectors. At the base of the fold the IP domain is predominantly red (Supplementary Figure S6.2c), whereas it is dominated by the green/blue sectors at the peak of the fold (Supplementary Figure S6.2d).

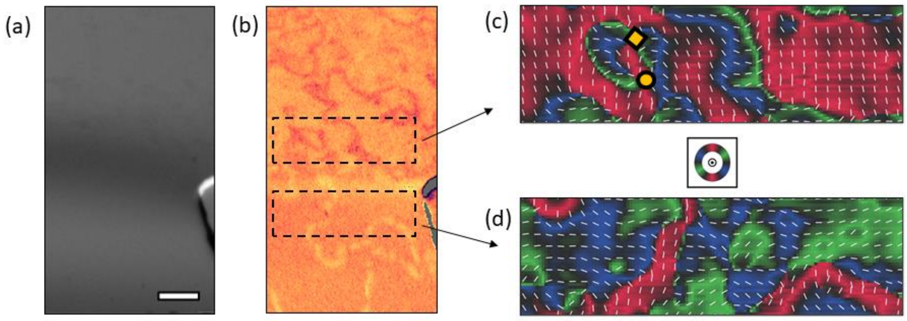

Supplementary Figure S6.2: Evidence of IP domain reconfiguration. (a) Fe L_3-edge X-ray transmission contrast obtained using STXM. The spatial scale bar is 2 μm. **(b)** AFM STXM contrast obtained at $T > T_{\mathrm{M}}^{\mathrm{FF}}$. **(c,d)** AFM vector-maps obtained at the fold's (b) base and (c) peak. R-G-B colours and thin white bars indicate IP orientations. R-G-B colours and thin white bars represent the IP AFM orientations.

S7: Strain simulations

Finite element strain simulations were performed to estimate the flexural strains resulting from the microscale 'folding' of the membranes (see Methods). The simulation was optimised to match the shape of the experimental structure obtained using confocal microscopy, see Supplementary Figure S7.1a. In principle, there are 3 non-vanishing tensor elements needed to fully describe the strain across the membrane fold, the diagonal terms, ε_{xx}, ε_{zz}, and the off-diagonal term, ε_{xz}. Note that z direction is along the membrane thickness, whereas x, y lie along the membrane such that y is the unstrained direction. As illustrated in Supplementary Figure S7.1b-e, we find that the dominant strain term is ε_{xx}, whereas the ε_{zz} and ε_{xz} terms are smaller by about 1- and 2 orders-of-magnitude, respectively. Hence, only the ε_{xx} term has been used in the estimation of strain-induced changes to the Morin transition in main text Figure 4.

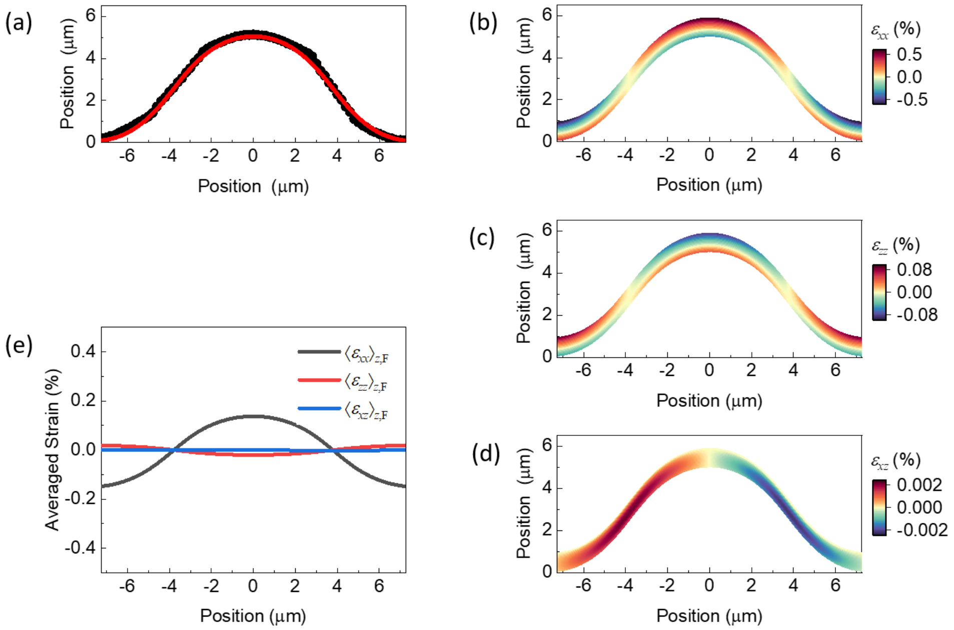

Supplementary Figure S7.1: Further details on strain simulations. (a) Comparison of the experimental (black) and simulated (red) cross-sectional shape of the membrane fold shown in main text Figure 3. **(b-d)** Non-uniform spatial variation of the strain tensor elements (b) ε_{xx} (c) ε_{zz} and (d) ε_{xz} in the α-Fe_2O_3 layer as a function of thickness and length, across folds in buffered AFM membranes. Membrane thickness has been exaggerated to aid the visualization of the non-uniform strain distribution as a function of thickness. (e) Evolution of the thickness averaged strain tensor elements in the α-Fe_2O_3 layer calculated using the data in (b-d).

S8: Dependence of the Morin transition on strain

The Morin transition results from a delicate temperature-dependent competition between the magnetic dipolar and on-site anisotropy interactions in α-Fe_2O_3. These interactions have opposite signs such that they favour easy-plane and easy-axis spin configurations, respectively. The interactions vary with lattice parameters as they are sensitive to the position of the Fe^{3+} cations in the lattice. Therefore, strain changes the relative strength of the two interactions, thereby enhancing and suppressing the T_{M} for compressive and tensile strains, respectively (see Supplementary Figure S8.1).[S5] Moreover, the effect of the in-plane strain was found to be 1 order of magnitude larger than that of the out-of-plane strain.[S5] Hence, in our study, we only focus on the effect of ε_{xx}.

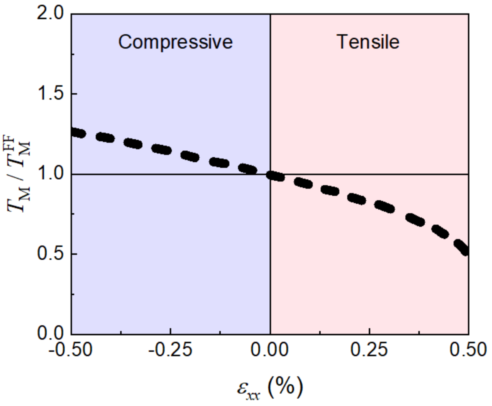

Supplementary Figure S8.1: Dependence of the Morin transition in the strained region relative to the flat region as a function of compressive and tensile strains, based on the model in Ref [S5]. At zero strain $T_{\mathrm{M}} = T_{\mathrm{M}}^{\mathrm{FF}}$ (black vertical line in the centre). It should be noted that Ref [S5] reports the strain dependence of the function $T_{\mathrm{M}}/T_{\mathrm{N}}$, which is multiplied with $\left(T_{\mathrm{N}}^{\mathrm{FF}}/T_{\mathrm{M}}^{\mathrm{FF}}\right) \times \left(T_{\mathrm{N}}/T_{\mathrm{N}}^{\mathrm{FF}}\right)$ to convert it to $T_{\mathrm{M}}/T_{\mathrm{M}}^{\mathrm{FF}}$. The quantity in the first bracket is a constant whereas that in the second bracket is the strain dependence of T_{N}, found to be linear and very close to 1 from the theoretical calculations in Ref [S13].

S9: Comparison of experimental and modelled AFM state distribution

To validate the efficacy of our mechanical model presented in Supplementary-S7, we compare the temperature-dependent distributions of the AFM states determined from the experimental STXM images across the flexed region (main text Figure 3) to their counterparts calculated from the strain-driven modulation of the local T_M (main text Figure 4). To do this, we determine the IP AFM fraction across the fold, which is a position-dependent variation of the ratio $\mathrm{Area_{IP}}/(\mathrm{Area_{IP}} + \mathrm{Area_{OOP}})$, as illustrated below in Supplementary Figure S9.1. We find that the experimental and modelled trends of the IP AFM fraction are generally consistent within the experimental error margin, suggesting that the model reasonably reproduces the experimentally observed AFM state reconfiguration ensuing from microscale flexure.

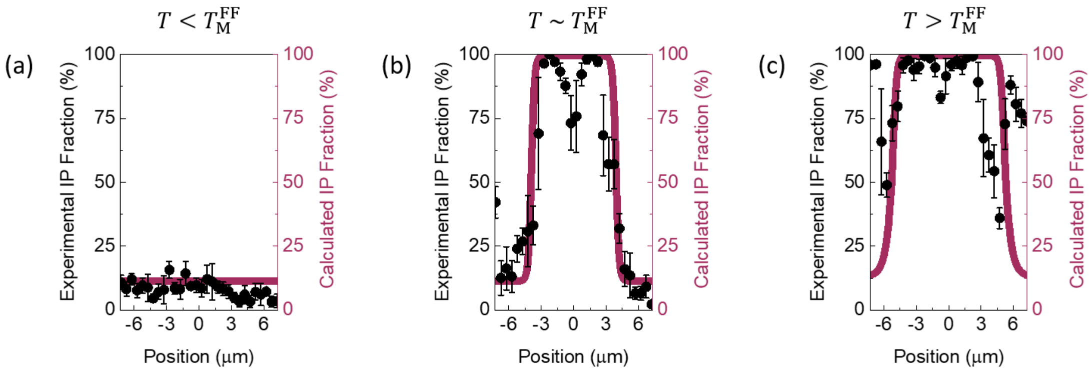

Supplementary Figure S9.1: Comparison of the *experimental* (black circles) and *modelled* (purple lines) AFM state distribution across the fold, corresponding to the sample temperatures **(a)** $T < T_M^{FF}$, **(b)** $T \sim T_M^{FF}$, and **(c)** $T > T_M^{FF}$. Spatial variation of the modelled IP fraction (purple lines) was determined using the strain driven modulation of the local T_M, see the mechanical model in main text Figure 4c. The peak of the fold is located at the zero position in each figure. The experimental fraction of the IP AFM states was determined from the XMLD STXM images shown in main text Figure 3. To do this, we exploited the fact that each STXM image contains both OOP and IP AFM regions which produce extreme XMLD contrasts. A threshold intensity, calculated as the average contrast, was then used to produce a binary filter. The IP fraction is the fraction of the total pixels with values greater than the threshold. The IP fraction is 50% when the membrane is in the middle of the Morin transition. Note that, below the local T_M, about 0-20% IP fraction is still present due to the IP regions in the ADWs. Alternatively, above the local T_M, the IP fraction is in the range of 80-100% due to the presence of OOP cores or pinned regions. Error bars correspond to the standard deviation of the experimental IP fraction inside a box of 10 pixels.

S10: Structural defects and moiré pattern in AFM membranes

Crystalline growth of the α-Fe_2O_3 layer on the sacrificial SAO or buffer layers and the STO substrates results in the formation of structural defects, such as dislocations, due to the sizable lattice mismatch. This is evident from the high-resolution TEM (HR-TEM) imaging performed on the membranes, see Supplementary Figure S10.1. These results are fully consistent with previous HR-TEM experiments performed in attached α-Fe_2O_3 films grown on STO (111).[S14] The inter-defect length scale is typically <100 nm, which is much smaller than the distance between topological textures shown in main text Figures 2, 5. Therefore, while defects can in general play a role in pinning topological textures, see Supplementary-S2, they cannot be responsible for generating them.

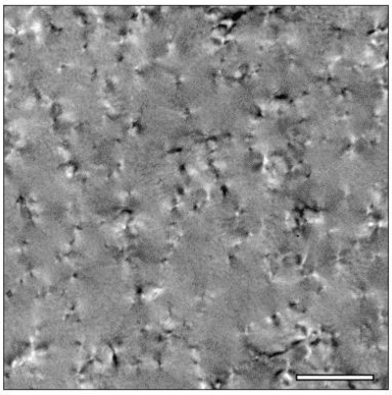

Supplementary Figure S10.1: Low magnification HR-TEM image reveals the presence of structural defects, such as dislocations, which appear as regions of dark/bright contrast in the grey background of type-B membranes. The spatial scale bar is 200 nm long.

Next, the HR-TEM experiment also confirmed the formation of a near-hexagonal spatial moiré pattern in our buffered membranes, see Supplementary Figure S10.2, which is justified based on the symmetry of the α-Fe_2O_3 and the buffer layers. The distortion of the moiré pattern results presumably from the presence of structural defects formed during growth.

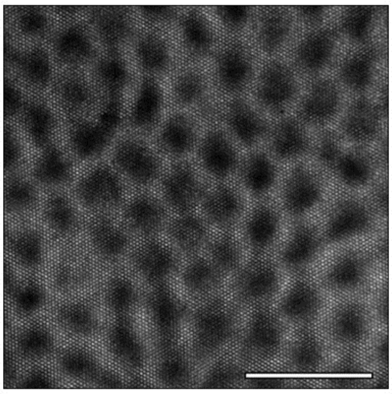

Supplementary Figure S10.2: High magnification HR-TEM image reveals the existence of a spatial moiré pattern as the origin of the satellite peaks observed in the SAED images of Supplementary Figure S1.1e and Figure 1e, taken in type-C membranes. The spatial scale bar is 10 nm long.

S11: AFM image processing steps

In the following, we discuss the steps required to generate the vector-maps from XMLD STXM images. Angle-dependent STXM images were first rotated and drift-corrected using common non-dichroic spatial features such as cracks or extraneous defects. These images were then cropped to a consistent size, removing any areas that fell outside at least one of the images, see Supplementary Figure S11.1. FFT bandpass filtering was employed to remove minor horizontal stripes, if any, that may come from the line scanning nature of the technique. Next, a polynomial correction was performed to remove any background, followed by Gaussian blurring to reduce the experimental noise between neighbouring pixels. Finally, a pixel-wise fit of the angular intensity was performed to a $\cos^2\psi$ function, where ψ is the relative angle between the linearly polarised X-ray electric field and the magnetization, to produce the vector-map, see Supplementary Figure S11.1b and Methods. Theoretical details supporting this approach can be found in Methods and Ref [S1,S15,S16]. It is evident from Supplementary Figure S11.1a that whilst yellow-orange regions (corresponding to IP orientation) change with rotation angle, the purple regions (OOP orientation) remain predominantly unchanged. This is expected because OOP AFM domains remain invariant under rotation about the crystallographic 3-fold axis, validating our assignment of IP vs OOP AFM orientation.[S1]

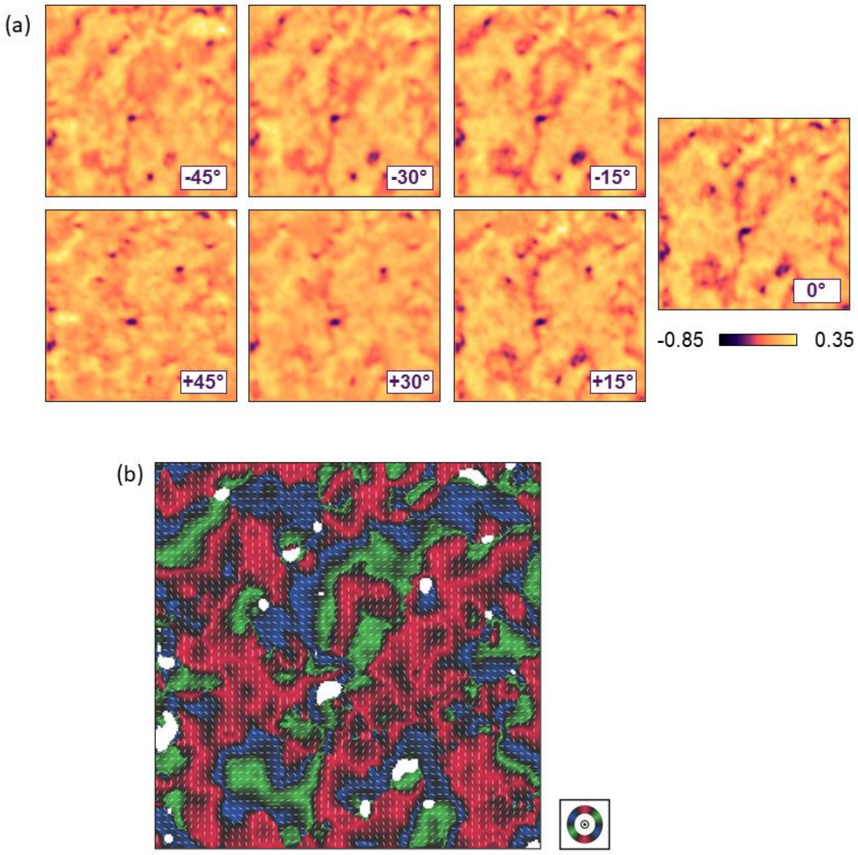

Supplementary Figure S11.1: Example of the vector-map construction process. **(a)** Single XMLD images obtained by rotating the sample angle relative to the X-ray polarisations between -45° and +45°, at the same position. The OOP and IP contrasts are indicated in purple and yellow/orange, respectively. The size of each image is ~ 5.6 µm × 5.6 µm. Although the image processing steps mentioned above, consistently applied to all images used for vector-mapping, can shift the absolute value of the XMLD energy contrast, this does not affect the vector reconstruction itself because the relative changes between images remain preserved. **(b)** Fully reconstructed vector-map from which several cut-outs were taken to highlight topological textures in main text Figure 2. R-G-B colours and thin white bars represent the IP AFM orientations. White regions represent OOP orientations, whereas black regions highlight IP AFM directions deviating substantially from the R-G-B directions.

S12: Temperature correlation

Although there is generally good qualitative correspondence between the AFM evolution observed in magnetometry and STXM imaging, the temperature dependence has a minor difference in the two, due to the different thermal properties of supporting layers and any small strain variation introduced during the corresponding sample preparation steps. Hence, in the following we contrast the thermal evolution of the IP domain fraction measured using STXM experiments against the IP canted moment measured in SQUID magnetometry. We find an upward shift of ~7.5 K is required to better correlate the STXM and magnetometry temperature values, see Supplementary Figure S12.1.

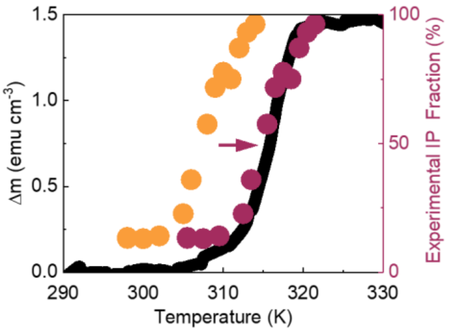

Supplementary Figure S12.1: STXM temperature correction factor. We compare the temperature evolution (during the warming sequence) of the IP domain fraction measured via the AFM STXM contrast in main text Figure 2b (orange circles) against the evolution of the canted magnetism measured during the warming sequence in Supplementary Figure S1.3c (black line). An upward shift of ~7.5 K (purple circles) results in better reproduction of the magnetometry data. Note that the IP fraction is 50% when the membrane is in the middle of the Morin transition. Below T_M, about 0-20% IP fraction is still present due to the IP regions in the ADWs.

References

[S1] Jani, H. *et al.* Antiferromagnetic half-skyrmions and bimerons at room temperature. *Nature* **590**, 74 (2021).

[S2] Jani, H. *et al.* Reversible hydrogen control of antiferromagnetic anisotropy in α-Fe_2O_3. *Nature Communications* **12**, 1668 (2021).

[S3] Moriya, T. New Mechanism of Anisotropic Superexchange Interaction. *Physical Review Letters* **4**, 228 (1960).

[S4] Dzyaloshinsky, I. A thermodynamic theory of "weak" ferromagnetism of antiferromagnetics. *Journal of Physics and Chemistry of Solids* **4**, 241 (1958).

[S5] SeongHun, P. *et al.* Strain control of Morin temperature in epitaxial α-Fe_2O_3 (0001) film. *Europhysics Letters* **103**, 27007 (2013).

[S6] Legrand, W. *et al.* Room-temperature stabilization of antiferromagnetic skyrmions in synthetic antiferromagnets. *Nature Materials* **19**, 34-42 (2020).

[S7] Pylypovskyi, O. V. *et al.* Curvilinear One-Dimensional Antiferromagnets. *Nano Letters* **20**, 8157-8162 (2020).

[S8] Makarov, D. *et al.* New Dimension in Magnetism and Superconductivity: 3D and Curvilinear Nanoarchitectures. *Advanced Materials* **34**, 2101758 (2022).

[S9] Pavlis, A. & Psaroudaki, C. Curvature-induced skyrmion mass. *Physical Review Research* **2**, 032058 (2020).

[S10] Carvalho-Santos, V. L., Corona, R. M., Altbir, D. & Castillo-Sepúlveda, S. Shifts in the skyrmion stabilization due to curvature effects in dome- and antidome-shaped surfaces. *Physical Review B* **102**, 024444 (2020).

[S11] Yershov, K. V., Kákay, A. & Kravchuk, V. P. Curvature-induced drift and deformation of magnetic skyrmions: Comparison of the ferromagnetic and antiferromagnetic cases. *Physical Review B* **105**, 054425 (2022).

[S12] Sheka, D. D. *et al.* Nonlocal chiral symmetry breaking in curvilinear magnetic shells. *Communications Physics* **3**, 128 (2020).

[S13] Kota, Y., Imamura, H. & Sasaki, M. Strain-Induced Néel Temperature Enhancement in Corundum-Type Cr2O3 and Fe2O3. *Applied Physics Express* **6**, 113007 (2013).

[S14] Tao, A. *et al.* Single-Dislocation Schottky Diodes. *Nano Letters* **21**, 5586-5592 (2021).

[S15] Chmiel, F. P. *et al.* Observation of magnetic vortex pairs at room temperature in a planar α-Fe_2O_3/Co heterostructure. *Nature Materials* **17**, 581 (2018).

[S16] Waterfield Price, N. *et al.* Coherent Magnetoelastic Domains in Multiferroic $BiFeO_3$ Films. *Physical Review Letters* **117**, 177601 (2016).
